# Diagnostic image quality of hysterosalpingography: ionic versus non ionic water soluble iodinated contrast media

**DOI:** 10.2349/biij.5.3.e29

**Published:** 2009-07-01

**Authors:** H Mohd Nor, KJ Jayapragasam, BJJ Abdullah

**Affiliations:** Department of Biomedical Imaging, University of Malaya, Kuala Lumpur, Malaysia

**Keywords:** Hysterosalpingography, contrast media

## Abstract

**Objective:**

To compare the diagnostic image quality between three different water soluble iodinated contrast media in hysterosalpingography (HSG).

**Material and method:**

In a prospective randomised study of 204 patients, the diagnostic quality of images obtained after hysterosalpingography were evaluated using Iopramide (106 patients) and Ioxaglate (98 patients). 114 patients who had undergone HSG examination using Iodamide were analysed retrospectively. Image quality was assessed by three radiologists independently based on an objective set of criteria. The obtained results were statistically analysed using Kruskal-Wallis and Mann-Whitney U test.

**Results:**

Visualisation of fimbrial rugae was significantly better with Iopramide and Ioxaglate than Iodamide. All contrast media provided acceptable diagnostic image quality with regard to uterine, fallopian tubes outline and peritoneal spill. Uterine opacification was noted to be too dense in all three contrast media and not optimal for the assessment of intrauterine pathology. Higher incidence of contrast intravasation was noted in the Iodamide group. Similarly, the numbers of patients diagnosed with bilateral blocked fallopian tubes were also higher in the Iodamide group.

**Conclusion:**

HSG using low osmolar contrast media (Iopramide and Ioxaglate) demonstrated diagnostic image qualities similar to HSG using conventional high osmolar contrast media (Iodamide). However, all three contrast media were found to be too dense for the detection of intrauterine pathology. Better visualisation of the fimbrial outline using Ioxaglate and Iopramide were attributed to their low contrast viscosity. The increased incidence of contrast media intravasation and bilateral tubal blockage using Iodamide are probably related to the high viscosity.

## INTRODUCTION

Hysterosalpingography (HSG) refers to the radiographic evaluation of the uterine cavity and fallopian tubes after injection of a radio-opaque contrast medium through the cervical canal. It is commonly and the initial investigation for evaluating fallopian tube disorders associated with infertility [1]. Despite recent advances in various imaging modalities to assess the fallopian tubes such as a three-dimensional dynamic magnetic resonance hysterosalpingography (3D dMR-HSG) and contrast enhanced hystero-salpingo-sonography, conventional HSG still remains the imaging modality of choice [2]. HSG was first performed by Rindfleisch in 1910 when he injected Bismuth solution into the uterine cavity [3]. In 1925, Heuser used an oil soluble medium, Lipiodol to demonstrate the uterine cavity [4]. Thereafter, oil soluble contrast media became the contrast media of choice for the next 50 years. However, its popularity decreased as there were reported adverse effects such as pulmonary oil embolism, acute tubal blockage, re-activation of tubal infection and granuloma formation. These complications were reduced with the introduction of water soluble contrast media. Water soluble contrast media achieved popularity for HSG in the 1960s and 1970s as it was associated with good radiographic quality and less serious side effects [5]. The initial water soluble contrast media was hyperosmolar (> 1000 mosmol/kg) and ionic accounting for most of the side effects [6].

Enormous efforts have taken place to develop safer contrast media while maintaining good diagnostic image quality. In 1985, these efforts led to the development of non-ionic low osmolar water soluble contrast media such as Iopramide (Schering, Berlin, Germany) in Europe [7]. Previously, large controlled studies have mainly compared diagnostic image quality between iodinated oil-based and water soluble contrast media in HSG. Only a few large controlled studies have been undertaken to compare the diagnostic image quality of low osmolar contrast media based on their ionic/non-ionic component and viscosity.

Accordingly, this study was performed to compare the diagnostic image quality of the three types of water soluble iodinated contrast media; hyperosmolar ionic contrast medium Iodamide (Bracco, Italy), low osmolar ionic contrast medium Ioxaglate (Guerbet, France) and low osmolar non ionic contrast medium Iopramide for HSG. These water soluble contrast media have different physical properties. The characteristics of the contrast media are listed in Table 1

**Table 1:** Chemical and Physical properties of the Contrast Media.

Properties	Iodamide	Ioxaglate	Iopramide
Chemical Nature	Ionic water soluble	Ionic water soluble	Non Ionic water soluble
Iodine Contents (mgI/ml)	249	320	300
Viscosity (mPa.sec, 37°C)	150	7.5	4.6
Osmolality of a 280mgI/ml (mosmol/kg)	1500	490	470

## PATIENTS AND METHODOLOGY

A prospective, randomised, double-blind study was designed to compare the diagnostic image quality between low osmolar ionic contrast medium Ioxaglate and low osmolar non-ionic contrast medium Iopramide for HSG on 204 patients. These are the total number of patients who had undergone HSG between August 2006 and September 2007. The 204 patients were randomly assigned to receive either Iopramide (106 patients) or Ioxaglate (98 patients). 114 patients who had undergone HSG study using Iodamide, which is a hyperosmolar ionic contrast medium, between August 1998 and September 1999 were analysed retrospectively. This was because the production of this contrast medium was stopped in year 2000, hence prospective analysis could not be carried out. All these patients were referred for HSG in the authors' department because of primary or secondary infertility. Patients who have undergone unilateral salpingo-oophrectomy and those with history of pelvic discharge (pelvic inflammatory disease), recent dilatation and curettage, possible pregnancy and patient with history of contrast media allergies were not included in this study. The patient’s age and race were recorded for equal distribution of these contrast media. All patients completed a consent form before undergoing the procedure. Patients did not know which contrast medium was used. The protocol was approved by the ethical research committee at the authors' institution.

The examination was performed between day 7 and day 10 of menstrual cycle or in patients who have abstained from sexual intercourse since their last menses.

A 5 French (F) hysterosalpingo (HS) foley balloon catheter was used for cervical cannulation. Leech Wilkinson cannula was used in the retrospective study in those patients who received Iodamide. Patients received no pre-medication. The radiologists who performed the procedures were unaware of the type of contrast medium used. Under fluoroscopic guidance, 5-10 ml of the contrast medium warmed to body temperature was injected until free peritoneal spillage occurred or tubal obstruction was demonstrated. In cases of tubal spasm the examination was repeated after 5 minutes following intravenous administration of 10 milligram of hyoscine butylbromide (Buscopan).

All radiographs were acquired using high kilovolts technique (90-120kV) adjusted by automatic exposure control. Three standard views were obtained: one frontal (when the contrast medium filled the uterus) and two obliques (when peritoneal spillage or presumed tubal blockage was seen during fluoroscopy). The images were printed on hardcopies using standard windowing.

Three radiologists blinded to the contrast media used independently assessed the images based on an objective set of criteria. The uterine cavity opacification referred to the uterine contrast density. The uterine cavity, fallopian tube and free peritoneal spillage outline were graded based on their sharpness. As for the fimbriaes, assessment was made based on the ability to see the longitudinal rugations of the ampullary portion of the fallopian tubes. A 4-point scoring system was applied for image analysis. All the images were scanned through to select the reference images for the scoring system which were agreed upon by the three radiologists. Diagnostic quality was considered excellent (score 3) when there was good opacification and clear delineation of the analyzed structure was achieved. Radiographs were classified as poor (Score 1) when there was poor contrast opacification but anatomic delineation and visibility were adequate for diagnosis. Anything better was given a score of 2. Radiographs were deemed unacceptable (Score 0) when the opacification and the delineation of the anatomical structure interfered with diagnosis. Interpretation discrepancies were resolved by consensus discussion.

The images were analysed and results were tabulated. Non-parametric independent samples test (Kruskal-Wallis) was used to statistically evaluate the obtained results. When significant difference was noted, non-parametric independent Mann Whitney U test was then used to see which of the two contrast media led to the difference. A p value of less than 0.05 (p<0.05) was considered significant.

## RESULTS

No significant differences were observed between the three contrast media groups with regard to age and race distribution. These findings are summarised in Table 2. Radiographic imaging quality was considered good to excellent with no significant differences (p > 0.05) between the three contrast media with regard to uterine outline, fallopian tube outline and free peritoneal spillage outline. However, visualisation of fimbrial rugae was significantly better (p <0.05) with Iopramide and Ioxaglate than Iodamide. In some cases of tubal disease, the tubes, fimbrae, and peritoneal distribution were not visualised, thus the scoring was not possible and these cases were not included in the study. Uterine opacification was noted to be excellent but too dense in all three contrast media. The results from the evaluation in terms of diagnostic image quality are summarised in Table 3.

**Table 2:** Comparison of patients' data in the Iodamide, Iopramide and Ioxaglate groups.

Contrast Media	Iodamide	Iopramide	Ioxaglate
Patients	114	106	98
Race:			
-Malay	57 (50%)	67 (63.2%)	63 (64.3%)
-Chinese	23 (20.2%)	16 (15.1%)	12 (12.2%)
-Indian	32 (28.1%)	21 (19.8%)	22 (22.4%)
-Others	2 ( 1.8%)	2 ( 1.9%)	1 ( 1%)
p = 0.055 (Race)			
Age (years):			
-mean	30.5	30.17	30.83
-range	21 – 44	20 – 45	21 – 49
p = 0.104 (Age)			

**Table 3:** Scoring of image quality in different anatomical regions of interest using three different contrast media.

Anatomical / Ratings	Contrast Medium			
	Iodamide	Iopramide	Ioxaglate	p value
Number of patients	114	106	98	
Uterus opacification				
0	0 ( 0%)	0 ( 0%)	0 ( 0%)	
1	0 ( 0%)	0 ( 0%)	0 ( 0%)	1.000
2	2 ( 1.8%)	2 ( 1.9%)	1 ( 1%)	
3	112 (98.2%)	104 (98.1%)	97 (99%)	
Uterine outline				
0	0 ( 0%)	0 ( 0%)	0 ( 0%)	
1	0 ( 0%)	0 ( 0%)	0 ( 0%)	1.000
2-3	114 (100%)	106 (100%)	98 (100%)	
Fallopian Tube				
0	0 ( 0%)	3 ( 2.9%)	1 ( 1.0%)	
1	15 (13.3%)	20 (19.2%)	14 (14.3%)	0.173
2-3	98 (86.7%)	81 (77.9%)	83 (84.7%)	
Fimbrial rugae				
0	11 ( 9.7%)	7 ( 6.7%)	5 ( 5.1%)	
1	31 (27.4%)	19 (18.3%)	17 (17.3%)	<0.05
2-3	71 (62.8%)	78 (75.0%)	76 (77.6%)	
Intraperitoneal spillage				
0	0 ( 0%)	0 ( 0%)	0 ( 0%)	
1	3 ( 3.1%)	0 ( 0%)	0 ( 0%)	0.05
2-3	94 (96.9%)	101 (100%)	91 (100%)	

Summarized results from the ratings of image quality, 0 = Non diagnostic, 1=Acceptable Quality and 2-3 = Superior Quality. Statistical calculations are based on raw data

All the patients involved in this study had a score of 2 and above for demonstration of uterine outline regardless of which contrast media was used. No differences existed among the three water soluble contrast media.

As for the fallopian tubes, the image quality was good to excellent in all contrast media with a score of 2 and above (86.7% with Iodamide, 77.9% with Iopramide and 84.7% with Iodamide). There were 3 patients in the Iopramide group with non-diagnostic fallopian tube outline (Score 0). No patient had a score of 0 in the Iodamide group. Even though no significant difference was noted, the scoring of the fallopian tube was slightly better in the Iodamide group.

The scoring of the fimbrial rugaes showed an evident difference amongst the three contrast media. Only 62.8% of the Iodamide group were rated 2 and above in comparison to 75% and 77.6% of the Iopramide and Ioxaglate group respectively. This difference, even though small, was statistically significant (p <0.05). Further analysis was carried out to investigate which of the two contrast media led to the differences by using a non-parametric independent Mann Whitney U test. There was a significant difference between Iodamide and Iopramide and also between Iodamide and Ioxaglate. Iopramide and Ioxaglate showed comparable scores for the fimbrial rugae outline.

The image quality of the peritoneal spillage was not significantly different. Majority of the scorings were high with 96.9% of the Iodamide and 100% of both Iopramide and Ioxaglate showing ratings of 2 and above.

With regard to contrast opacification of the uterine cavity, all the patients involved in this study had good to excellent scores. The uterine cavity opacification refers to the uterine contrast density. It was found that 98.2% patients from the Iodamide group, 98.1% patients from the Iopramide group and 99% patients from the Ioxaglate group had excellent scores but were too dense for diagnosing intrauterine pathology. The remaining had a score of 2.

The number of patients with both unilateral and bilateral blocked tubes were 37% in Iodamide, 29.3% in Iopramide and 31.6% in Ioxaglate and statistically, no significant differences exist. However, when the three contrast media were re-analysed with regard to the diagnosis of bilateral blocked tubes alone, there were significant differences noted (p< 0.05). There were 14.9% of patients from the Iodamide group who demonstrated bilateral blocked tubes in comparison to only 4.7% from Iopramide group and 7.1% from Ioxaglate group. These findings are summarised in Table 4.

**Table 4:** The incidence of tubal blockage using three different contrast media.

Diagnoses	Contrast Medium		
	Iodamide	Iopramide	Ioxaglate
Unilateral Tubal Block	20 (17.6%)	26 (24.6%)	24 (24.5%)
Bilateral Tubal Block	17 (14.9%)	5 ( 4.7%)	7 ( 7.1%)

Numbers represent number of blocked tubes.

The numbers in brackets is the equivalent in percentages.

Contrast intravasation was the only immediate complication which was recorded and analysed. 19 patients had demonstrated contrast intravasation, of which 18 of them had lymphatic intravasation and 1 patient had venous intravasation. Of these 19 patients, 15 of them had their examination performed with Iodamide and 4 patients used Iopramide. There was a significant difference in the incidence of contrast intravasation between the three contrast media. The incidence of contrast intravasation with bilateral blocked tubes in these 19 patients was further associated. Of the 15 patients who received Iodamide, 7 patients had blocked tubes while the other 8 patients had normal tubes. Similarly, there was equal incidence of blocked and normal tubes in the 4 patients who used Iopramide. None of these 19 patients were diagnosed with hydrosalpinx. The incidence of contrast intravasation into the lymphatic and venous system is shown in Table 5.

**Table 5:** Incidence of contrast media intravasation using three different contrast media.

Contrast	Contrast Intravasation	Blocked Tubes	Patent Tubes	Hydrosalpinx
Iodamide	15 (13.2%)	7	8	0
Iopramide	4 ( 3.8%)	2	2	0
Ioxaglate	0 ( 0%)	0	0	0

Numbers represent number of contrast intravasation. The numbers in brackets is the equivalent in percentages.

## DISCUSSION

HSG is mainly performed to assess radiographic signs of peritubal adhesions such as convoluted tubes, vertical tubes, loculation of contrast medium in peritoneum, fixed laterodeviation of the uterus and congenital uterine abnormalities. An ideal contrast agent for HSG should provide excellent radiopacity to delineate the uterine cavity and tubes and should be non-toxic and non-irritating to endometrial, tubal, and peritoneal surfaces. Oil-based contrast media was not included in this study as the outcome of the study may not have contributed to the future. Lindequist et al. had performed two large prospective randomised studies in 1991 and 1994 comparing oil-based and water soluble contrast media with regard to diagnostic image quality and concluded that both water soluble and oil-based contrast media are comparable when it comes to uterine opacification, fallopian tube and free peritoneal spillage outline. Fimbriae and uterine outline are not as well demonstrated by oil-based ethiodised poppy seed oil due to its high viscosity (25mPa.s, at 37 0 Celsius) [5, 8].

In this study all the three water soluble iodinated contrast media demonstrated comparable and excellent image quality of the uterine cavity, fallopian tubes and free peritoneal spillage, And no significant differences exist between the three contrast media . The natural ability of these water soluble contrast media to mix with the watery mucous surface within the uterus probably contributes to the excellent visualisation of this structure [5].

**Figure 1:**
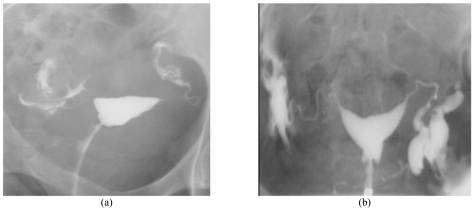
Demonstration of fallopian tube outline with Iopramide (a) and Iodamide (b). Note the sharper outline of the fallopian tubes seen in the Iodamide group in comparison with Iopramide group.

Adequate viscosity is required to prevent such rapid filling of the uterine cavity leading to excessive spillage into the peritoneal cavity before radiographs are obtained. The demonstration of the fallopian tubes were slightly better in the Iodamide group due to its higher viscosity which raises its transit time within the fallopian tube thus allowing enough time before the radiograph is obtained. Iopramide, having the lowest viscosity among the three studied contrast media, flows rapidly through the fallopian tubes (short transit time) thus does not stay long enough within the fallopian tubes before the radiograph is obtained. This explains why there were more patients in the Iopramide group that had non-diagnostic fallopian tubes (Score 0) in comparison to Iodamide (Figure 1).

**Figure 2:**
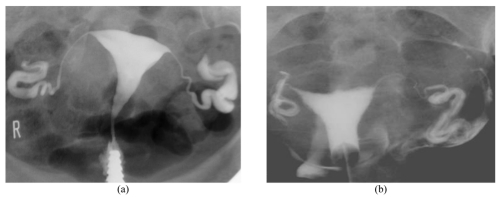
Demonstration of fimbrial rugae outline with Iodamide (a) and Iopramide (b). Note the sharper outline of the fimbrial rugaes seen in the Iopramide group in comparison with Iodamide group.

Although the three contrast media demonstrated fairly good visualisation of the fimbrial rugaes, an evident difference was seen between the three contrasts. Good and clear visualisation of the fimbrial rugaes were required in order to grade the image quality as good or excellent which was in favour of Iopramide and Ioxaglate (Figure 2). It is possible that the high viscosity of the Iodamide prevents proper coating of the mucosal surface of the fimbrial rugaes thus leading to its poor visualisation. Previous studies comparing fimbrial rugae outline using low viscosity water soluble contrast media and high viscosity ethiodised-poppy seed oil also showed poor demonstration with the latter contrast media [5, 8].

**Figure 3:**
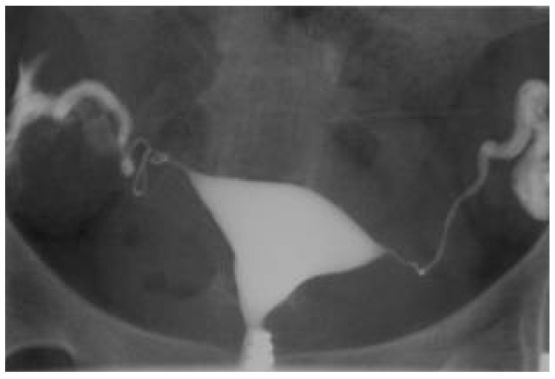
This is a HSG study image using Iodamide contrast media (conventional contrast media). Note the uterine cavity is too dense for the visualisation of filling defects from an intra-uterine submucosal fibroid.

The radiopacity depends primarily on the percentage of organically bound iodine in each contrast medium. Iodamide contains 249 mg of organically bound iodine while Iopramide and Ioxaglate contain 300 mg and 320mg I/ml respectively (Table 1). Contrast media with an iodine content of 200-300 mg/ml produce adequate density for diagnostic images. The iodine concentration of all the three contrast medium is high. Therefore, it is not surprising that all the three contrast media provided sharp image of the uterine cavity with great contrast density as others have demonstrated. These results are also comparable with previous studies [5, 8]. Even though the three contrast media demonstrated superior opacification of the uterine cavity, they were too dense for the detection of intrauterine pathology (Figure 3). The uterine cavity opacification refers to the uterine contrast density. An excellent score for uterine contrast opacification does not necessarily mean it is ideal for the detection for uterine pathology. Ideal uterine cavity opacification refers to the ability of uterine contrast density to detect intrauterine pathology without obscuring them. The ability to see the posterior uterine wall was the criteria that used to determine ideal uterine cavity opacification. This assessment showed that 98.2 to 99% of the patients from all three contrast media showed uterine contrast opacification that was too dense for the detection of intra-uterine pathology. Siegler et al. had indirectly emphasised that point in his study. He commented on the danger of overfilling the uterine cavity with contrast material when evaluating the shape of the uterus in search of submucosa myomas, endometrial polyps, or synechiae. He said that increasing amounts of contrast material can obscure the classic findings of a submucosa myomas seen as a filling defect of a distorted, enlarged cavity [9]. Thus, based on the findings of this study, further studies are required for optimising the diagnostic quality of uterine opacification, hence to improve the diagnostic potency of intra-uterine pathology and further reducing false negative result.

**Figure 4:**
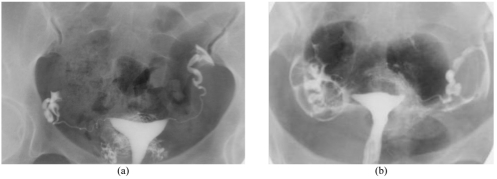
Note the lymphatic contrast intravasation obtained with Iodamide (a) and Ioxaglate (b). None of the patients showed immediate symptoms.

The number of patients with both unilateral and bilateral blocked tubes was almost similar amongst the three contrast media. However, a significant difference was evident in the diagnosis of bilateral blocked tubes which were in favour of Iodamide. The diagnosis of bilateral blocked tubes were 2-3 times higher in the Iodamide group in comparison to Iopramide and Ioxaglate groups. The sample size of this study is large and the contrast media were randomised among the patients. Thus the detection of bilateral blocked tubes should be roughly the same. It is assumed that the increased detection of bilateral blocked tubes in the Iodamide was attributed to its higher viscosity. The higher viscosity of Iodamide prevents smooth flow through the fallopian tubes causing it to pool within the tubes which led to the apparent blockage of the fallopian tubes (false positive). This group of patients would have probably demonstrated patent tubes if Iopramide or Ioxaglate, which have lower viscosity, were used instead. Previously, many studies have shown the potential therapeutic effect of oil-based contrast media post-HSG in achieving pregnancy [8, 10-12]. Many hypotheses were postulated as to the therapeutic effect of the oil-based contrast media which might more effectively flush out tubal plugs involved with proximal tubal blockage. Goodman et al. hypothesised that peritoneal lymphocyte proliferation and macrophage phagocytosis were significantly inhibited by ethiodol which may have contributed to the increased post-HSG pregnancy rate [12]. It is postulated that the previously believed therapeutic effect of the oil-based contrast media may not be actually true and the patients involved probably did not have completely blocked tubes in the first place. Thus the clinical importance of the findings of this study findings questionable. To the knowledge of the authors, the effect of low viscosity water soluble contrast media on the apparently blocked tubes has not been investigated and such investigations are warranted.

This study showed that patients from the Iodamide group recorded higher number of contrast intravasation in comparison to Iopramide and Ioxaglate. Further analysis of this group of patients with contrast intravasation showed that there were equal numbers of blocked and normal tubes implying that the contrast intravasation is not just related to blocked tubes. The increased viscosity of Iodamide inadvertently resulted in excessive pressure during the injection of contrast material. Our results are comparable to that of previous studies [13]. Venous and lymphatic contrast intravasation was considered a serious side effect in HSG in the past, causing pulmonary embolism. This risk was reduced with the use of low viscosity oil-based and water soluble contrast media. None of the patients with water soluble contrast intravasation developed immediate pulmonary or anaphylactoid symptoms. In a larger series, embolic complications were not reported at all [5]. The infrequent incidence of complications from a practical standpoint seems to be a minor problem.

**Figure 5:**
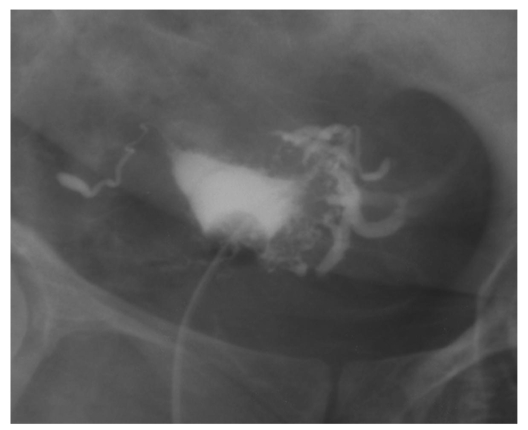
Venous intravasation seen as a network of thin vessels on top of uterus and in a pattern that could be confused with tubal filling.

Radiographically, early contrast intravasation appears as filling of multiple thin beaded channels following an ascendant course. Although venous and lymphatic channels can often be identified by their anatomy, contrast intravasation into uterine and ovarian veins can sometimes be mistaken for tubal filling. This becomes a disadvantage and results in false positive results (Figure 5)

This study did have some limitations. The HSG examination was not done by the same investigator. Several authors have shown the importance of consistency in injection pressure and spot film timing in carrying out HSG procedures. Differences in both of these aspects have been shown to have a great effect on visualisation of anatomic contours as well as the assessment of tubal patency, filling and contrast intravasation. In addition to this, Leech Wilkinson cannula was used in HSG during the era of Iodamide. Thus, the appearance of Leech Wilkinson cannula on the HSG hard copies gave a clue to the radiologist on the type of contrast media used when interpreting the results, hence causing biasness in reader reliability. Besides, there was no variety of pathology seen in this study to suggest the adequacy of the density of contrast media. Contrast opacification of the uterus needs re-evaluation regarding optimal technique and contrast viscosity. Hence it is proposed that further studies are carried out for optimising the diagnostic quality of uterine opacification.

## CONCLUSION

Low osmolar contrast media (Iopramide and Ioxaglate) had diagnostic image quality comparable to the conventional high osmolar (Iodamide). No significant difference was found regarding density of uterine opacification, delineation of the uterus and fallopian tubes, or spillage into the peritoneal cavity. Better visualisation of the fimbrial outline using Ioxaglate and Iopramide was attributed to the low contrast viscosity. The increased incidence of bilateral tubal blockage and contrast intravasation are probably related to the high contrast viscosity. All three contrast media were too dense for the detection of intra-uterine pathology and needs further evaluation.
